# Carbohydrate Knowledge and Expectations of Nutritional Support among Five Ethnic Groups Living in New Zealand with Pre- and Type 2 Diabetes: A Qualitative Study

**DOI:** 10.3390/nu10091225

**Published:** 2018-09-04

**Authors:** Zhuoshi Zhang, John Monro, Bernard J. Venn

**Affiliations:** 1 Department of Human Nutrition, University of Otago, PO Box 56, Dunedin 9054, New Zealand; zhuoshi.zhang@gmail.com; 2 New Zealand Institute for Plant & Food Research Ltd., Private Bag 11600, Palmerston North 4442, New Zealand; John.Monro@plantandfood.co.nz

**Keywords:** diabetes, ethnicity, knowledge, discussion groups, qualitative

## Abstract

Despite availability of diabetes and nutrition information for people with pre- and type 2 diabetes, the uptake and understanding of these resources may differ among ethnic groups. Our objective was to explore dietary knowledge and diabetes experiences amongst Māori, European, Pacific Island, Indian and East Asian people living in New Zealand with a focus on carbohydrate-containing foods. A registered diabetes dietitian led ethnic-specific discussions in groups involving 29 people with pre- or type 2 diabetes. Discussions were audio-recorded, fully transcribed and coded independently by two investigators. Themes were developed using deductive and inductive techniques. Five themes emerged: knowledge, concerns, achievements, simplicity and self-determination. Nutritional knowledge was lacking and a greater awareness of trustworthy dietary resources was needed. There were concerns about diabetes complications and appropriate carbohydrate-containing foods and portions. Contrary to this, people felt proud when achieving dietary goals and grateful for support from health care providers and family. Participants were willing to engage in self-care if advice from health professionals was given in plain language, and in a culturally appropriate manner. Given the desire to take an active role in diabetes self-management and willingness to use electronic devices, an ethnic-specific nutrition education resource could be a valuable tool.

## 1. Introduction

Diabetes is one of the fastest growing chronic conditions in the world [1,2,3]. In 2014, an estimated 422 million adults were living with diabetes compared to 108 million in 1980 [4]. To provide the best diabetes care, a structured multidisciplinary team input is required [5,6]. However, the reality for many countries is a general lack of resources to cater for the growing number of people with diabetes, resulting in a knowledge gap for people both recently diagnosed and for those with a longstanding diagnosis [7,8]. To fill the knowledge gap, people with pre- and type 2 diabetes can obtain information on their condition from healthcare professionals and also from less regulated sources such as the internet or from other people [9]. However, information obtained from unregulated sources can lead to misunderstanding, frustration and anxiety [10,11,12], poor compliance in treatment [12,13,14,15] and unnecessary food avoidance [12,16,17]. In questioning people with diabetes, there was confusion about the effect of macronutrients on glucose metabolism with carbohydrates being particularly misunderstood to the extent that some people were avoiding fruit due to the sugar content [17]. Psychological stresses for people with diabetes including fear, worry and perceived discrimination have been found in the second Diabetes Attitudes, Wishes and Needs (DAWN2) study [10].

Negative emotions and lack of knowledge could be even more pronounced in ethnic minorities living in Caucasian counties, who tend to receive less diabetes education [18], have less diabetes and nutritional knowledge [19], are less engaged with diabetes services [20,21], and have higher emotional distress [22,23] compared with the ethnic majority. Compounding these inequalities is a tendency for ethnic minority groups to have a higher prevalence of type 2 diabetes and to have poorer health outcomes compared with the ethnic majority group [18,24,25].

Identifying barriers and expectations to diabetes care and nutrition education is important if ethnic inequalities are to be addressed. Little work has been carried out comparing differences among ethnicities regarding diabetes nutrition management [10,12,13,17]. New Zealand is a country in which ethnic experiences can be explored because it has indigenous and immigrant minority groups living in a predominantly Caucasian population, reflecting ethnic diversity in many other countries [26]. The research described herein was aimed at exploring experiences and emotions among Māori, European, Pacific Island, Indian, and East Asian people living with diabetes or pre-diabetes using ethnic-specific discussion groups. The purpose was to ascertain participants knowledge and beliefs pertaining to diabetes and nutrition management with an emphasis on carbohydrate-containing foods, and expectations of diabetes care.

## 2. Materials and Methods

### 2.1. Ethics and Recruitment

Discussion groups were recruited through verbal referral from general practitioners’ practices, primary health organisations and community health support services; and via advertisements in local medical centres, libraries, community centres, sports facilities and supermarkets within Auckland city, New Zealand. A Māori group was recruited in Palmerston North, New Zealand through contacts with funders of the study. Adults with pre- and type 2 diabetes who expressed interest were asked for their consent to receive a telephone call regarding the study. Eligible participants were invited to attend one discussion group based on their ethnicity. The inclusion criteria were: A diagnosis of pre-diabetes or type 2 diabetes confirmed by medical records, New Zealand residency and the ability to communicate in English. The exclusion criteria were people with severe speech or hearing difficulties, inability to speak English or over 79 years of age. The Ngāi Tahu Research Consultation Committee was consulted and the study was approved by the University of Otago Human Ethics Committee (Reference no. 14/179).

Of the 71 referrals and respondents (19 Europeans, 10 Māori, 11 Pacific Islanders, 14 East Asian and 17 Indian), 13 people could not be contacted and 22 declined. Of the remaining 36 people confirmed, seven did not show up on the day. Thus, 29 participants (six Europeans, five Māori, four Pacific Islanders, eight East Asians and six Indians) attended separate ethnic-specific discussion groups. Prior to the discussion group meetings, an information sheet was given to each participant, queries were answered and consent was obtained.

### 2.2. Procedures

The discussion groups were designed and conducted in accordance with a published protocol [27]. The discussions were held onsite in Auckland and via Skype^®^ in Palmerston North with Māori participants, a method of synchronous interviewing that is becoming more widely used [28]. The duration of each discussion group was approximately 1 h, and was conducted in a safe and comfortable environment with each session audio-recorded. An interviewer guide was used to facilitate each discussion. 

Predefined questions were open-ended and designed to avoid wording suggestive of a ‘correct’ answer. All participants were encouraged to speak freely, whilst the facilitator (Z.Z.) ensured that the discussion moved at an appropriate pace and finished on time. Sentence completion exercises and brainstorming on a whiteboard were used during the discussion. At the end of the session, the purpose of the discussion was repeated and final questions from participants were answered. The predefined questions were as follows:

- What do you know about pre-diabetes and type 2 diabetes?
- What diabetes support have you received?
- What nutrition for diabetes advice have you been given by clinicians?
- Where do you seek for diabetes and nutrition information and how accurate is it?
- What do you want to know about nutrition for diabetes?
- Have you seen a dietitian for pre- and type 2 diabetes?
- What foods do you think affect your blood glucose?
- What foods do you think are healthy or unhealthy?
- Would an electronic diabetes nutritional education resource be useful for you?

### 2.3. Data Analysis

A published thematic approach involving deductive and inductive techniques was used [29]. A priori, the broad code categories of knowledge, experience and desire were selected. All audio recordings were transcribed verbatim by the facilitator (Z.Z.) and checked by a second investigator (B.J.V.). Photographs were taken to record notes written on a whiteboard during discussions. Two researchers (Z.Z. and B.J.V.) independently coded the transcript. Potentially important words and phrases were identified through both inductive and deductive analysis. These two sets of coding phrases were compared. Any discrepancies were discussed through reviewing of the transcript and the meaning of a code until consensus was reached. Coding phrases were further developed into themes through creating a coding manual.

## 3. Results

Participant characteristics are given in Table 1. The average time since diagnosis for the nine people with pre-diabetes was 2.6 years and for those with type 2 diabetes, 13.7 years. Of the 29 participants, six participants were not on any diabetes medication. Of those on medication; 19 were prescribed metformin; eight sulfonylureas; one dipeptidyl peptidase-4 inhibitor and four used insulin with or without oral medications.

### 3.1. Knowledge

General practitioners (GPs) and registered nurses (RNs) were identified as the two main sources providing diabetes dietary advice across all discussion groups. Half of the participants felt that they had not received adequate nutrition advice. Being unaware of, and having limited access to a diabetes service was emphasised within the Māori, Pacific Island and Indian discussions. All groups voiced a strong need for adequate consultation time with health professionals to discuss their queries and concerns regarding nutrition and diabetes control.

*“You don’t really have a lot time, you go in there, and you just don’t have time to ask some questions.”—European group*

*“So far the lecture from diabetes centre was the only one come and discuss something (sic). We have not heard from anybody else. It will help a long way if there are more dietitian lectures.”—Indian group*

Apart from three people who had recently attended diabetes education classes, participants struggled when asked to describe pre- and type 2 diabetes, what the risk factors were of developing type 2 diabetes, and the reasons for pharmaceutical and lifestyle intervention. European, Māori and East Asian groups commented on the inconsistency of medical and nutritional information obtained from the Internet, and of more concern, among health professionals. Confusion due to lack of diabetes knowledge or being exposed to conflicting information was apparent within all ethnic groups. Animated discussions on suitable food and beverage options, and how to interpret laboratory test results, occurred in all discussions.

*“You go on to the Internet and you can find that this is good. Do this and do that, and you can also go to another parts and it says this is all wrong.”—European group*

Identifying “good” and “bad” dietary choices was another key discussion point in all discussions. Most participants were restricting foods and beverages that were perceived by them to be bad for diabetes whilst increasing the intake of so-called good foods. Although all participants understood foods and drinks with added sugar increased blood glucose concentrations, the majority failed to recognise or understand that other carbohydrate in food has blood glucose raising potential.

*“Eat less potatoes, we are not allowed to eat potatoes, only once a week.”—Indian group*

*“These three fruits are deadly for diabetes. They are very high in sugar.”—Māori group*

The foods in Table 2 were identified within the groups as raising blood glucose concentration. Foods identified as raising blood sugar among all groups were sugar and rice. Bread was not specifically referred to by any group and only the Indian group mentioned potato. Some ethnic-specific starchy foods were identified; taro by the Pacific Island group and noodles by the East Asian group. Three out of four Pacific Island and half of the European and East Asian participants believed that fatty foods raised blood glucose. In conclusion, half of the participants across all ethnic groups considered that they had received inadequate nutrition information from the health system. Conflicting messages from various official, online and lay sources led to misunderstanding, confusion and unnecessary dietary restriction. Māori, Pacific Island and Indian participants were less likely to have accessed specialist diabetes and dietetic services in both primary and secondary care.

### 3.2. Concerns

All participants were fearful and worried about diabetes complications, medication side effects, eating unhealthy foods and having inappropriate portion sizes. Suspicion regarding the credibility of information obtained from health professionals and other sources was expressed by all except the Indian group who relied heavily upon the information provided by general practitioners and practice nurses. The Indian participants were less aware of specific diabetes education services compared with the other groups. A lack of involvement in diabetes management decision making left the majority of Māori and Pacific Island participants feeling powerless over their own health destiny. One Māori and one East Asian participant also described feelings of embarrassment in discussing their diabetes with friends and family.

*“How do you know that we got diabetes? Because I don’t know I have diabetes, until I had a stroke.”—Pacific Island group*

*“It says it affect the heart, kidneys, eyes and foot. We’ve told it starts with eyes, heart, kidney and sensation of the foot. Sensation of the foot starts lose, any disease on the foot is difficult to get cured.”—Indian group*

Participants of all ethnicities, except Indian, expressed aversion to a top-down nutrition consultation style in which health providers were viewed as judgemental. With regard to food, there was some confusion and resentment expressed in all groups with health care providers making incorrect assumptions, giving mixed messages, and imposing unexplained dietary restrictions.

*“The practice nurse assumed that I drank a lot of juice and coke. And I said, No! I don’t!”—Māori group*

*“My GP tells me the same thing, keep doing some exercise, eat these and don’t eat that. But I said, the food is just food. What you just told me not to eat is not fair.”—Pacific Island group*

*“What about apple? Red apple or granny smith? Just one apple? What about rock melon? Yoghurt?”—Indian group*

*“My GP asked me to go back to Korean diet like rice and soup. I am kind of confused, cause I eat so much rice.”—East Asian group*

*“You tend to not like suddenly a whole lot of restrictions coming from middle of nowhere, telling you that you can’t eat this bread roll, you can’t drink this, you can’t do this, you can’t do that. And you rebel.”—European group*

Frustration was expressed that some of the nutrition advice was conflicting both among health care providers and between the advice received and what they had read or believed. One Pacific Island and one Indian participant were angry with themselves for relapsing into unhealthy eating habits and not paying enough attention to their own health. 

Concern was expressed in the Māori, Pacific Island and Indian groups regarding self-adjustment of medication and treatment. The main drivers were doubt about their understanding of best practice and fear of diabetes complications.

*“I started to feel shaking of my hands. Then I take some sugar or any foods. Once I okay with it, I stop. If I am outside, the best thing I do is buy bananas, two or three bananas, and one or two lollies.”—Indian group*

*“Knowing that the insulin should have not been starting at two units. I raised it up myself to straight up to 20, 30 units. My sugar level was too high and I can sense things going wrong in the eyes. I’ve just started to deal with it myself.”—Māori group*

In summary, participants were fearful about diabetes complications. Doubt, confusion, embarrassment, powerlessness, frustration and anger were all negative emotions expressed by different ethnic groups.

### 3.3. Achievements

All ethnic groups stated an interest in understanding diabetes, diabetes medications, diet, lifestyle, and how to make changes. Several participants in the European, Māori and Pacific Island groups expressed strong desires to halt the progression of diabetes and to stay healthy. Several participants from each ethnic group described feeling satisfied and even delighted when achieving goals such as achieving recommended blood glucose and maintaining dietary change. Some participants described their sense of gratitude towards families, friends, healthcare professionals, and other people with diabetes for psychosocial and medical support. Although this was mentioned in all group discussions, appreciation of family involvement was specifically highlighted by the majority of the Māori and Pacific Island participants.

*“Sometimes, I get frustrated. I didn’t want to take any more medicine, but my wife talk me out of it. That’s why I need my family, because they are the part taking care of you when you are at home.”—Pacific Island group*

Recognising the need for good diabetes control and the setting of life goals, such as preventing diabetes complications and spending quality time with families, served as inspiration. Many participants expressed satisfaction from having discovered ways to change diet and lifestyle, being able to sustain these changes, avoiding temptation, and ultimately improving blood glucose control. Although the Indian group expressed determination to make change, the sense of achievement conveyed by the other groups was absent from the Indian group discussion.

*“I felt really good now. I said I feel I have to do something for myself, and I will see a real change, even up until now, I am a really changed person.”—Pacific Island group*

*“You need to have a reason to want to live in a long healthy life. For me, I want to see my grandchildren.”—East Asian group*

*“I realise in the end it is the weight. I just concentrated on the quantity that I eat, and when I eat. I stopped the night snacking. I love chocolate, but I can now go to the fridge and look at the chocolate and then just walk away.”—European group*

Overall, all ethnic groups showed positive attitudes such as feeling motivated and satisfied when good diabetes control was attained. Family support was a highlight for Māori and Pacific Island groups.

### 3.4. Simplicity

All groups wanted simple explanations regarding appropriate food choices. A pictorial or video format illustrated with ‘hands’ and ‘plate’ models were favoured over text-based advice, particularly if text incorporated scientific jargon. Some European, East Asian and Indian participants were also receptive to advice in numerical form for ease of compliance, for example, 30 min of exercise.

*“Dietitian said I could have enough potatoes like 3 small eggs, so that’s how I used to measure my carbohydrate portions. It’s like 3 small egg size.”—East Asian Group*

*“Plate is plate. Half, quarter, quarter simple (for vegetables, protein and carbohydrate).”—Māori Group*

Participants wanted advice to be practical, especially around home cooking methods and dietary patterns. Participants discussed how simple, practical advice that they had received had increased their confidence in trying and maintaining dietary and lifestyle changes. Being culturally appropriate was also highlighted by East Asian and Pacific Island groups. Although European, Māori and Indian groups felt comfortable with English-language-based education, being able to receive information in their own language was preferred by some participants in East Asian and Pacific Island groups.

*“With diabetes, I want to know how to cook from what you have in your cupboards rather than buy all these lovely things, which is not realistic for your diabetes.”—Māori group*

*“Beans, corn, and nuts… See we’ve never eaten these good foods. We weren’t brought up with it.”—Pacific Island group*

*“It will be nice if you can speak Samoan.”—Pacific Island group*

All ethnic groups expressed a desire for simple, visual and practical dietary advice. Some participants wanted dietary advice to include cultural foods and to be presented in their familiar language.

### 3.5. Self-Determination

Māori, Pacific Island and Indian participants expressed a willingness to make lifestyle changes but felt overly dependent on treatment plans assigned by health professionals. Several participants described frustration at the lack of involvement in decision-making around their own diabetes care, citing poor communication with their healthcare provider. All groups indicated reluctance in making dietary and lifestyle changes because they felt inadequately informed as to the need for change as well as perceived difficulties in avoiding temptation and fitting additional self-monitoring tasks into their life.

*“It’s more peace of mind, actually explaining what the medication is, instead of, go it’s one of these.”—Māori group*

*“The chemist said, Oh! You don’t need this medication anymore. It is not on the prescription. You are on this. I turned around and said, what do you mean? It isn’t inside of what I have been taking? They’ve changed that, and I never knew.”—Pacific Island group*

Most participants supplemented the advice of their health professionals by taking initiatives to improve their diabetes knowledge by reading books, searching online, attending diabetes classes, making dietary changes and self-monitoring blood glucose.

*“You would look it on the computer, because the information is there.”—Māori group*

*“I read all about the diabetes myself from library books.”—East Asian group*

All ethnic groups wanted to have ongoing support and reminders to achieve and maintain a healthy diet and to increase compliance with medication. Peer support was valued by several Māori, Pacific Island and European participants who found sharing their experiences with other people with diabetes helpful and reassuring.

*“Diabetes, diet is the main issue. Required to be reminded it again and again.”—Indian group*

*“Being around with people got the same illness as myself, it’s like a support group (sic). What they said was exactly what I am going through. And I think, none of us has been perfect. We all did the same thing.”—Pacific Island group*

The use of new technology to search for health-related information and recipes, and to create meal plans was clearly shown in all discussions. Convenience and immediate information feedback were highly regarded attributes, although reliability of this information was a major concern. Only five out of 29 participants said they would probably not use online learning resources, with cost and age identified as barriers.

*“When we went on the Internet, we have to depend on it. Sometimes there is different information on one topic.”—Indian group*

*“You cut back on the portions of your meal and then finish with a piece of fruit. You don’t really know if it is the right thing or not. If you have a website that you could look at would be quite good.”—European group*

## 4. Discussion

The main findings of the discussion groups were a recognised lack of knowledge and confusion regarding diet and medication, fear of diabetes complications, a willingness to participate in the management of their condition, the need to keep advice practical, the use of simple language, avoidance of medical jargon, and engagement with self-directed searching for information despite some level of mistrust and confusion with Internet sites. All ethnic groups expressed willingness to modify diet and carbohydrate sources given reliable and culturally appropriate guidance. Ethnic-specific issues included Māori, Pacific Island and Indian participants discussing lack of group education and access to specialist diabetes services. Explanation as to why dietary modification and medication treatment was needed would provide motivation for change in the Māori and Pacific Island groups. Some Māori, Pacific Island and East Asian participants thought it desirable that information be made available in their own language. Some participants were self-checking blood glucose and body weight but there was a general feeling among the Indian participants that this should be monitored by clinicians rather than by themselves. The Māori and Pacific Island groups discussed family support more prominently than other ethnic groups.

One theme common to all groups was dietary restriction, a topic that was discussed with some resentment. In the United States, dietary restriction has been associated with diabetes distress, in part because it affected other members of the family [30]. To what extent carbohydrate foods should be restricted by people with type 2 diabetes is questionable, as carbohydrate intake has not been associated with long-term glycaemic control [31]. Food avoidance can be restrictive, but a positive idea that emerged from our Māori group was the desire to learn about food and nutrition in order to pass that knowledge onto the next generation. However, sourcing reliable dietary information was problematic for all groups. The European group cited ‘on-going referrals’ from health care providers to diabetes educational classes, whilst the other ethnic groups mentioned engagements in formal education sessions only sporadically. Our data are consistent with those obtained in Australia in which non-European ethnicity was associated with a lessened likelihood of having received formal diabetes education [18], highlighting the need to promote diabetes support services to non-European ethnic communities. 

Participants regarded the quality of diabetes information to be variable. The Internet was challenging as it provided instantly accessible information but conflicting messages. In other work it has been found that the quality of information is dependent on various factors including whether people are seeking factual information and advice on a course of action or subjective opinion [32]. The ‘hit’ rates for correct, complete or appropriate responses were low, typically around 50%, with lower scores for the advice type questions [32]. Hence, the frustration and confusion expressed by our participants is entirely consistent with other people’s experiences with the Internet [33]. The quality and safety of online diabetes information has been queried, with misinformation, promotional material, poor readability, lack of evidence-based recommendations and lack of medical disclaimers of concern [34,35,36]. Despite drawbacks, use of the Internet as a source of information will continue for all ethnicities as it is a resource that has broad reach and ease of access [37]. 

In contrast, there were differences among the ethnic groups for engagement and experience with health professionals. The European group discussed on-going interactions with healthcare services whereas Pacific Island participants seemed unaware of such services. The Indian group respected and trusted clinicians’ knowledge and judgment similar to British Indian patients [38,39] favouring clinicians’ guidance over self-care [40]. However, diabetes management requires a considerable component of self-care and advising Indian patients of the importance of this is essential [40]. The East Asian group reported positive experiences of healthcare providers despite lack of consultation time, reflecting experiences of Vietnamese women with gestational diabetes [41]. Our Māori participants were less trusting of the advice that they had received from health professionals. It has been documented that Māori and Pacific Island people are more likely to miss hospital appointments compared with Europeans [42]. Perhaps these sentiments and behaviours partly explain the reliance that Māori and Pacific Island participants placed on family for emotional and practical support, consistent with being more likely to live in multi-generational households and taking care of family members [43]. In South Africa, black African patients were found to be frequently receiving incorrect and inappropriate dietary advice from health educators [44]. In our participants, lack of dietary knowledge, confusion and mistrust in sources of dietary information identifies a clear need for reliable dietary education and advice. 

Cumulatively, the views expressed by our participants encompassed those found in Canada where limited consulting time, excessive workload and insufficient trust in physicians advice led to patients’ poor motivation and compliance [45]. It has been suggested that health systems set up to deal with acute care are poorly configured to cater to the needs of people with chronic conditions and that strategies are needed within those systems to foster self-management in people with diabetes [46]. A move away from an authoritarian approach to one of an equal partnership between health professionals and those with chronic conditions has been advocated [46]. Aspirational goals of partnership in disease management are in agreement with the views expressed by our participants who objected to a ‘top-down’ approach. To encourage partnership, communication with patients should be designed to answer their queries and to fit in with individuals’ goals and culture [46,47]. A patient-centred partnership program has facilitated patient participation and shared decision-making with health care providers [48]. Although trials designed to enhance patient-practitioner partnership may have positive outcomes in terms of patient engagement, more studies are needed to assess clinical outcomes following such intervention trials [49].

The present article describes novel research in which the experiences and expectations of diabetes management has been explored by a diabetes dietitian in participants representing five ethnic groups living in New Zealand. Sample numbers for each ethnic group were small and this poses a problem for generalisability within ethnicities as factors such as urban/rural; country of origin; cultural background; socioeconomic status; and education are underlying variables to the experiences and expectations of the individuals that participated. Nevertheless, the themes described herein were expressed in all discussion groups, suggestive that at least these themes may have commonality. In that context, the findings may have generalisability to other predominantly Caucasian countries whose population includes ethnic minority groups. In Australia, language, level of education and indigenous ethnicity were associated with lower diabetes knowledge and reduced likelihood of having received diabetes education and dietetic advice [18]. In the United States, immigrant and indigenous people experience diet-related disparities in which poorer diets are consumed by minority groups compared with the white populous [50]. Limitations to the generalizability of our study are that only participants who spoke English and who had an interest in diabetes management were involved. Potentially, our information is optimistic as people with a lesser command of the English language may struggle even more with accessing reliable information. Lack of knowledge of a country’s main language has been identified as disadvantaging access to health care [51]. Another limitation may be a small sample with participant numbers ranging from four to eight, although five has been suggested as an appropriate number for conducting group discussions [52]. Nevertheless, there are still limits on the generalisability of the discussions both within New Zealand, a geographically diverse country, and among other countries. Some issues though are likely to be common around the world, particularly with regard to ethnic minority groups living in Westernised countries. In summary, for people of various ethnicities with pre-diabetes and type 2 diabetes living in New Zealand, we have found heterogeneity in dietary knowledge and experience of the health care system. An overarching sentiment expressed by all ethnic groups was a desire for reliable dietary information to assist with self-management of the disease. 

Our participants expressed a need to access trustworthy sources of dietary information, a view consistent with previous work in which participants with pre- or type 2 diabetes were wary of some of the dietary information that they had accessed [53]. A general feeling was expressed that the nutrition advice of primary care providers was reliable although some reservations were expressed regarding inconsistency of messages and many participants supplemented the advice of their health professionals from other sources. Confusion around food and general dietary advice expressed by our participants is perhaps not surprising given that there is still uncertainty and controversy in the best dietary approaches for the prevention and management of type 2 diabetes [54].

It has been recommended that nutritional advice be given by a registered dietitian or by referral to a diabetes self-management education (DSME) program [55]. However, success in weight loss and diabetes remission over 1 year has been reported when participants were guided by general practitioners [56]. Nevertheless, it is unclear how well long-term nutrition goals and sustained weight loss can be maintained outside of the study setting, or whether it is feasible or necessary for continuing general practitioner involvement in dietary management. In a focus group survey of general practitioners practicing in Belgium, a sentiment expressed was that dieticians could give more adequate and more varied food advice than the general practitioners themselves [57]. Indeed, dietitian advice has been found to improve clinical outcomes in people with type 2 diabetes [58,59]. However, dietary advice is time-consuming and for many countries there are too few dietitians to adequately cater to the service demand [5].

## 5. Conclusions

It is telling that despite a plethora of electronic devices, websites and software applications, our participants struggled to find reliable information given in a practical and culturally appropriate manner. From the participants’ perspective, dietary advice needed to be consistent and to have been derived from a solid evidence base. With the challenge of an increasing prevalence of diabetes and limited health care resources, development of a professional organisation-endorsed, evidence-based, electronic resource using lay language, in video or pictorial format and available in different languages could be a highly sought after instructional tool. Consumer acceptance and performance of such a multi-ethnic resource would need to be monitored for efficacy.

## Figures and Tables

**Table 1:** Characteristics of the study participants.

	Total	European	Māori	PI	East Asian	Indian
	(*n* = 29)	(*n* = 6)	(*n* = 5)	(*n* = 4)	(*n* = 8)	(*n* = 6)
*N*, pre-diabetes	9	3	1		4	1
*N*, type 2 diabetes	20	3	4	4	4	5
Mean years of pre-diabetes	2.6	4.3	2.0		2.0	0.5
Mean years of type 2 diabetes	13.7	3.3	27.3	10.8	14.0	11.2
Sex, *n* (Male, Female)	11M, 18F	1M, 5F	3M, 2F	1M, 3F	2M, 6F	4M, 2F
Age, *n*						
45–54 year	3		1		2	
55–64 year	10	2	1	2	3	2
65–74 year	15	4	3	2	3	3
74–79 year	1					1
Diabetes medication, *n*						
None	6	3	1		2	
Metformin	19	3	3	3	5	5
Sulfonylureas	8		2	1	2	3
Insulin	4	1	2		1	
Other	1	1				
Education, *n*						
University	10	1			6	3
Polytechnic	7	2	2		1	2
Secondary	1	1				
Did not answer	11	2	3	4	1	1

PI, Pacific Islander.

**Table 2:** Foods thought by participants to affect blood glucose.

	Europeans	Māori	PI ^1^	East Asian	Indian
Carbohydrate foods or foods containing a substantial proportion of carbohydrate	Bakery food	Banana	Chocolate	Muffins	Beetroot
	Biscuits	Corn	Lollies	Fruit	Cola
	Cakes	Fruit	Rice	Instant noodles	Rice
	Chocolate	Refined sugar	Sugar	Rice	Soft drinks
	Pasta	Rice	Takeaways (e.g., sweet and sour)	Rice cakes	Potato
	Rice	Sprite	Taro	Sugar	
	Processed foods ^2^				
Foods containing little carbohydrate	Bacon		Fatty foods	Alcohol	Alcohol
	Butter			Cheese	
	Nuts				
	Sausages				

^1^ PI, Pacific Islander; ^2^ Processed foods may or may not contain substantial amounts of carbohydrate.
